# Can frailty in conjunction with FRAX identify additional women at risk of fracture - a longitudinal cohort study of community dwelling older women

**DOI:** 10.1186/s12877-022-03639-7

**Published:** 2022-12-09

**Authors:** Patrik Bartosch, Linnea Malmgren

**Affiliations:** 1 Department of Clinical Sciences Malmö, Clinical and Molecular Osteoporosis Research Unit, Lund University, 214 28 Malmö, Sweden; 2 Department of Orthopaedics, Skåne University Hospital, Malmö, Sweden; 3 Department of Geriatrics, Skåne University Hospital, 205 02 Malmö, Sweden

**Keywords:** (3–10) Frailty, Falls, Fracture, FRAX, Women, Community dwelling

## Abstract

**Background:**

Fracture risk assessment is still far from perfect within the geriatric population. The overall aim of this study is to better identify older women at risk for fractures, using a quantitative measure of frailty in conjunction with the web-based Fracture Risk Assessment Tool (FRAX®).

**Methods:**

This study was performed in the Osteoporosis Risk Assessment (OPRA) cohort of *n* = 1023, 75-year-old women followed for 10-years. A frailty index (FI) of ‘deficits in health’ was created, and FRAX 10-year probability for major osteoporotic and hip fractures was calculated and bone mineral density measured. Incident fractures were continuously registered for 10-years. Receiver Operating Characteristic (ROC) curves were used to compare FI, FRAX and the combination FI + FRAX as instruments for risk prediction. Discriminative ability was estimated by comparing Area Under the Curve (AUC). In addition, using guidelines from the Swedish Osteoporosis Foundation, a category of low risk women who would not have been recommended for pharmacological treatment (non-treatment group) was identified, categorized by frailty status and for relative risk analysis, hazard ratios (HR) and 95% confidence intervals were calculated using Cox proportional hazard regressions.

**Results:**

For hip fracture, FRAX and frailty performed almost equally (HIP AUC 10y: 0.566 vs. 0.567, *p* = 0.015 and *p* = 0.013). Next, FI was used *in conjunction* with FRAX; proving marginally better than either score alone (AUC 10y: 0.584, *p* = 0.002). Comparable results were observed for osteoporotic fracture.

In the non-treatment group (564 women), being frail was associated with higher 10y hip fracture risk (HR 2.01 (1.13–3.57)), although failing to reach statistical significance for osteoporotic fracture (HR 1.40 (0.97–2.01).

The utility of measuring frailty was also demonstrated when using T-score as an index of bone density to define fracture risk. Among *n* = 678 non-osteoporotic women, frailty added to the 10-year fracture risk (Hip; HR 2.22 (1.35–3.71); Osteoporotic fracture; HR 1.57 (1.15–2.14)).

**Conclusions:**

While the addition of frailty to FRAX marginally improved fracture prediction, applying a frailty measurement to a group of ‘low risk’ women, identified a set of individuals with high *actual* hip fracture risk that would not be prioritized for pharmacological treatment. Further cost-benefit analysis studies are needed to formally test potential benefit.

## Background

Given the current and expected demographic changes towards a growing population of older individuals [1], a specialized geriatric approach to medicine is essential to meet future healthcare demands. Frailty, encompassing the functional decline in multiple organ systems [2], is a central part of geriatric research and has been associated with multiple adverse health outcomes such as hospitalization and death [3, 4]. However, within frailty, the deterioration of the musculoskeletal system, affecting balance, mobility, falls and ultimately fractures [5–7], is perhaps the most dramatic. Indeed, frailty is not just a consequence of, but also a contributing cause of fracture. This vicious cycle of fracture and frailty can lead to further fractures and worsening frailty, in addition to a variety of adverse health outcomes [5, 8]. Given that fragility fractures already present a major health care burden, [9], and expected to increase, the association between frailty, osteoporosis and fragility fractures requires immediate attention.

One of the greatest problems in osteoporosis care is correctly identifying patients at high risk for primary fractures [10]. Despite the widespread use of Bone Mineral Density (BMD) assessment by Dual-energy X-ray Absorptiometry (DXA), a substantial number of women who subsequently suffer a fracture are not identified as being at risk since the majority do not have osteoporosis by definition [11]. This highlights that the fracture risk matrix is multi-factorial. Skeletal factors such as bone mass and strength are only one aspect, while non-skeletal factors play an increasing role with age, particularly with regard to hip fracture. As a clinically useful tool, incorporating multiple known risk factors, 10-year probability of fracture can be estimated using the web-based Fracture Risk Assessment Tool (FRAX®) algorithm [12]. However, defining high risk individuals is not entirely straight forward since guidelines differ nationally; different thresholds for FRAX are used [13] and ultimately clinical judgment from the responsible practitioner, weighing in all risk factors, will determine the treatment.

Further complicating the matter is that FRAX may underestimate fracture risk among those with a propensity to fall [14]. Indeed, past falls have been associated with incident fractures, independent of FRAX [15]. Of particular concern, is the fact that some individuals who are considered to be at low risk for fracture based on FRAX, and hence not prioritized for preventive fracture management, may be susceptible to falling as a consequence of higher frailty, with fracture as the probable outcome. Using only the FRAX algorithm to estimate fracture risk may therefore misclassify a large proportion of individuals, particularly geriatric patients that are also frail, resulting in substantial clinical implications. So while it is certainly good that FRAX is currently being implemented to estimate fracture risk in clinic, we think an extra layer is needed if we really want to capture as many ‘at risk’ individuals as possible, especially within primary fracture prevention.

With this background, the overall aim of this study was to obtain a clinical perspective on the utility of incorporating frailty into fracture risk assessment. Using the Osteoporosis Risk Assessment (OPRA) cohort of 1044 75-year old women, followed for 10 years, we therefore investigated whether using frailty in conjunction with FRAX provided a better risk stratification. In addition, to test our hypothesis that frailty could be of value in primary fracture prevention since it may capture non-skeletal risk factors, we measured frailty within a low risk group that would not be prioritized for treatment based on both FRAX and, as an index of bone density, T-score, and investigated fracture risk. Finally, appreciating the importance of BMD as a risk factor for future fractures, we examined if adding a frailty measure to those with low risk based on T-score alone could identify women at high risk of fracture.

## Materials and methods

### Subjects

This study uses the Osteoporosis Risk Assessment (OPRA) cohort of community dwelling women, all aged 75 year at baseline investigations. 1604 women were randomly selected to participate and invited by letter 1 week after their 75th birthday. 1044 women agreed to participate from 1995 to 1999 (65% response rate). No exclusion criteria was used. Follow-up investigations were performed at 5 years (*n* = 715, age 80.2 + 0.2) and 10 years (*n* = 382, age 85 + 0.1). At all three visits, extensive investigations were performed as previously described [16]. Detailed questionnaires provided supplementary information on lifestyle and health, while physical assessment (balance, gait, muscle strength, previous falls) and blood biochemistry were measured. This study uses only women with measurements for both FRAX and frailty, corresponding to 1023 women at baseline investigation.

The study was approved by the local ethics committee. Participants provided written informed consent. The study was conducted according to the principles of the Helsinki declaration.

### Frailty index

A frailty index (FI) was constructed using data collected at each visit according to the principles of Searle et al. [17]. Construction of the index is described in full elsewhere [18]. In brief, the index includes 13 variables covering a number of physiological domains (time spent outdoors, daily physical activity, balance, walking speed, number of steps taken, muscle strength, diabetes, cancer, diseases affecting balance, self-reported fall risk, polypharmacy, CRP and creatinine). The index represents the number of ‘deficits in health’, scored from 0.0–1.0; a higher score indicating higher frailty. This frailty index correlated very highly (*r* = 0.80) to a full 40-variable index [6], created for the two follow up visits, and both the 13- and 40-variable frailty index have similar ability to predict mortality [18].

### FRAX

Using the web-based Fracture Risk Assessment Tool, FRAX® (https://www.shef.ac.uk/FRAX/) [12] and baseline data (age 75), 10-year probability for major osteoporotic fractures and hip fractures was estimated. In this study, we analyse FRAX probabilities estimated *without* and *with* BMD at femoral neck included in the algorithm.

### Fractures, bone mineral density and T-score

Incident fractures were prospectively followed until October 2012 (up to 15 years) through the X-ray files at the Radiology Department, Malmö, Skåne University Hospital, as previously described [19]. Since the Department of Orthopaedics is the sole unit treating fractures in the catchment area, information loss during follow up is exceptionally low [19]. In this study we specifically analyse incident hip fractures and Major Osteoporotic Fractures (MOF) as defined by FRAX i.e. hip, vertebra, distal radius, shoulder. Fractures were analysed for a period of 10 years in order to compare with FRAX estimated 10 year risk. Fractures occurring prior to inclusion (i.e. below the age of 75) were registered, as previously reported [20].

BMD was determined using dual-energy x-ray absorptiometry (DXA) measured with a Lunar DPX-L (GE Lunar, Madison, WI), at femoral neck. The DXA machine automatically generated participants T-scores which, in standard deviations, show how much the measured BMD differs from the mean of a young population.

### Statistical analyses

Descriptive statistics are reported as mean and Standard Deviation (SD), median and Inter Quartile Range (IQR) or frequency and percentage, as appropriate. In case of non-normally distributed data, non-parametric analyses were performed when appropriate. The frailty index (FI) was stratified into quartiles (Q1 = lowest level of frailty; Q4 = highest level of frailty). Similarly, for FRAX (Q1 = lowest estimated risk of fracture; Q4 = highest estimated risk of fracture). Comparisons overall and between Q1 and Q4 were performed, as appropriate using ANOVA or Kruskal-Wallis tests. Correlation between the frailty index, FRAX_no BMD_ and BMD were tested using Spearman’s Rho.

To determine if frailty in conjunction with FRAX would enhance fracture identification, Receiver Operating Characteristic (ROC) curves were used to compare FI, FRAX and the combination FI + FRAX as instruments for risk prediction of fractures. The discriminative ability was estimated by comparing Area Under the Curve (AUC). Predictive power/goodness of fit of the models were confirmed using Harrell’s c statistic.

**Fig. 1:**
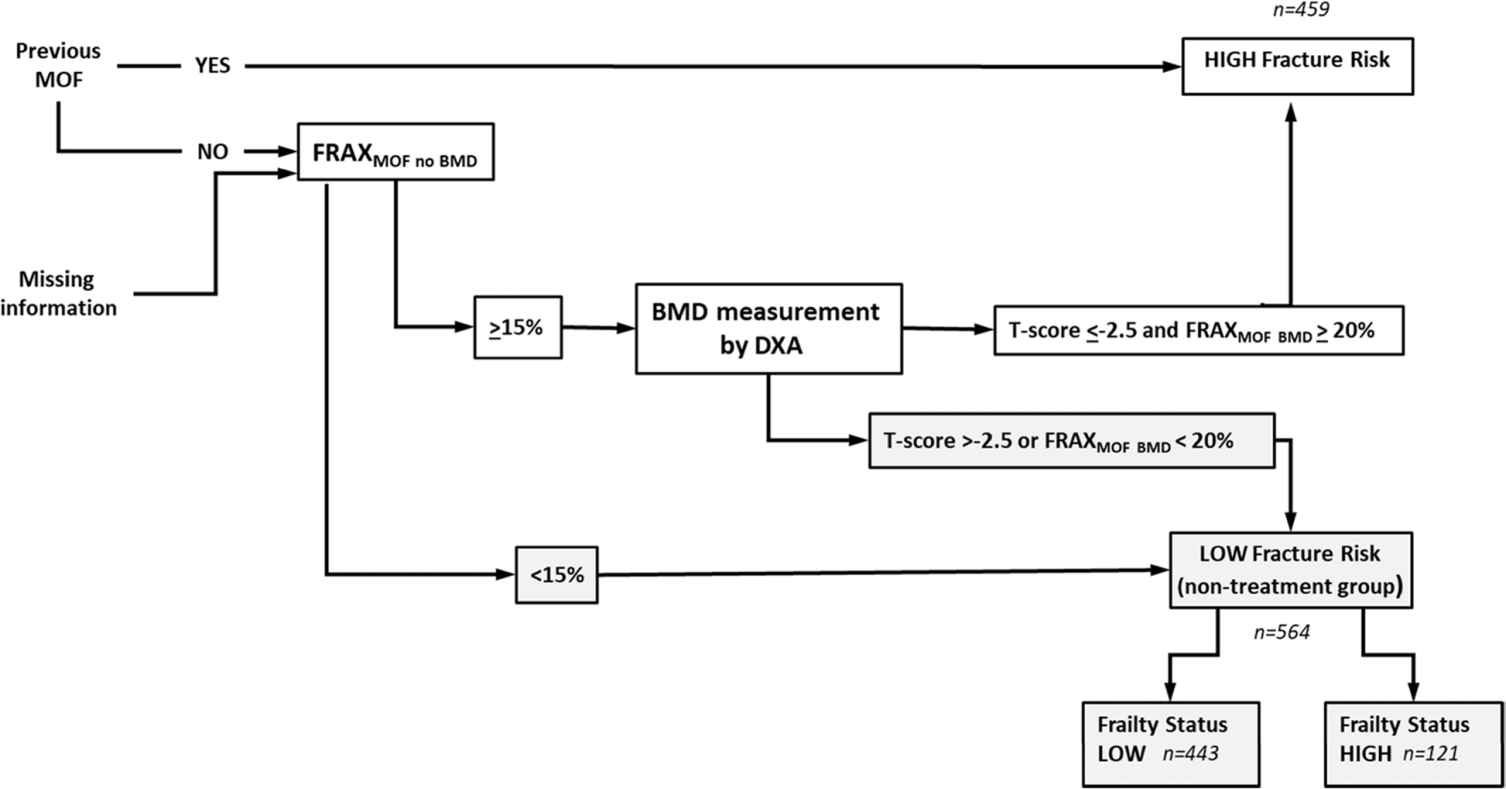
Selection of the non-treatment group. Data at age 75 was used to calculate FRAX_MOF_ probabilities. Women in the non-treatment group, i.e. who were ‘low fracture risk’ had no previous MOFs and either a FRAX_MOF_ < 15% or did not meet requirements for treatment after BMD measurement (i.e. T-score < − 2.5 and FRAX_MOF BMD_ > 20%). Low frailty includes Q1-Q3 and high frailty Q4

With the view of obtaining a clinical perspective on the utility of using frailty in primary fracture prevention to identify ‘at risk’ women who would not have would have been recommended for pharmacological treatment (i.e. non-treatment group), we first employed guidelines from the Swedish Osteoporosis Foundation [21] which uses estimates from the FRAX calculator in combination with measurements (BMD). As shown in the flowchart (Fig. 1), the 564 women in this category had no previous MOF and either a FRAX_MOF_ < 15% or did not meet requirements for treatment after BMD measurement (i.e. T-score < − 2.5 and FRAX_MOF BMD_ > 20%). This non-treatment group was then stratified by frailty index to determine how many subsequently suffered a fracture. To capture the frailest individuals we categorised FI < 0.27 as ‘low frailty’ (Q1-Q3) and ‘Highest frailty’ as FI > 0.28 (Q4). To investigate if frailty in conjunction with FRAX enhanced fracture identification within the non-treatment group, ROC curves were used to compare FI, FRAX and the combination FI + FRAX with AUC to determine discriminative ability in this subgroup of 564 women.

As a sensitivity analysis, and to investigate if frailty adds information about fracture risk beyond that of BMD alone, we also used the World Health Organization (WHO) definition, whereby a femoral neck T-score higher than − 2.5 [22] at age 75 was considered *low* fracture risk (*n* = 681). The *n* = 681 low risk women were then stratified by frailty index to determine how many subsequently suffered a fracture.

For relative risk analysis, hazard ratios (HR) and 95% confidence intervals were calculated using Cox proportional hazard regressions using the lesser frail quartiles (Q1–3) as reference category.

All analyses were performed using SPSS version 22 (SPSS, Inc., Chicago, IL) and JMP SAS (SAS Institute, Cary, NC, USA). *P*-values of < 0.05 were considered nominally significant.

## Results

The general characteristics of the OPRA cohort has been described in detail elsewhere, including fracture incidence [6]. For these 75 year olds, mean 10-year probability (%) of hip fracture using only FRAX was 13.8% (8.0) and 27.2% (10.0) for major osteoporotic fracture. We were interested in what characterized the women based on their different frailty status. This is shown in Table 1, presenting the main variables in this study according to quartiles of frailty at baseline (age 75). Predicted probabilities FRAX_HIP_ or FRAX_MOF_ (without BMD) did not differ between quartiles of frailty, nor did BMD or previous MOF (*p* > 0.50). As expected [6], women in the highest frailty quartile were more likely to have fallen within the previous year compared to those in the lowest frailty quartile (108 vs 31 falls, *p* < 0.001). Conversely, Table 2 shows the distribution of the frailty index, fractures and falls across quartiles of FRAX_MOF_. In contrast to categorisation by frailty index, falls, and even multiple falls did not differ across FRAX quartiles (*p* = 0.118 and *p* = 0.609), although the women with higher FRAX_MOF_ were more likely to have had a previous MOF (*p* < 0.001 for trend) and had lower BMD values (*p* < 0.001 for trend).

**Table 1:** Frailty by quartiles at age 75 and FRAX score, fractures and falls

All variables at 75y	Low Frailty Q1 (<=0.02)		Frailty Q2 (0.02–0.12)		Frailty Q3 (0.13–0.27)		Highly Frail Q4 (0.28+)		N^o^	p^#^ Overall	p Q1 vs Q4
	*n* = 261		*n* = 254		*n* = 262		*n* = 267				
	Mean	range	Mean	range	Mean	range	Mean	range			
**Frailty Index**	0.002	(0.00–0.02)	0.08	(0.03–0.12)	0.18	(0.13–0.27)	0.41	(0.28–0.88)		–	–
FRAX_HIP no BMD_	13.4	(5.1–61.0)	13.3	(4.3–57.0)	14.1	(4.2–53.0)	14.4	(3.9–58.0)	1023	0.362	0.172
FRAX_MOF no BMD_	26.3	(14.0–68.0)	26.7	(13.0–68.0)	27.7	(12.0–64.0)	28.1	(12.0–69.0)	1023	0.151	0.051
*Fractures*	N^o^	(%)	N^o^	(%)	N^o^	(%)	N^o^	(%)			
Prior any fx (20-75y)	97	(21.2)	104	(22.8)	122	(26.7)	134	(29.3)	457	0.011	0.002
Prior any fx (50-75y)	74	(19.3)	91	(23.8)	99	(25.8)	119	(31.1)	383	0.001	< 0.001
Prior MOF (50-75y)	55	(23.9)	53	(22.1)	65	(27.1)	67	(27.9)	240	0.505	0.242
*Falls in the last year* ^*1*^	N^o^	(%)	N^o^	(%)	N^o^	(%)	N^o^	(%)			
1 or more falls reported	31	(11.9)	43	(16.5)	78	(30.0)	108	(41.5)	260	< 0.001	< 0.001
3 or more falls reported	2	(2.9)	5	(7.2)	21	(30.4)	41	(59.4)	69	< 0.001	< 0.001
*Bone mineral density*^*2*^	Median	IQR	Median	IQR	Median	IQR	Median	IQR			
Femoral neck T-score	−1.78	(1.46)	−1.75	(1.60)	−1.98	(1.48)	−1.92	(1.56)	947	0.370	0.215
Femoral neck BMD (g/cm^2^)	0.766	(0.175)	0.751	(0.191)	0.742	(0.177)	0.749	(0.187)	947	0.370	0.215

Reported values are means, unless otherwise stated. ^#^ *p*-values calculated by ANOVA, Kruskal Wallace or Chi-square as appropriate

^1^Bartosch PS, Kristensson J, McGuigan FE, Akesson KE. Frailty and prediction of recurrent falls over 10 years in a community cohort of 75-year-old women. Aging Clin Exp Res. 2020;32 (11):2241–50

^2^Bartosch P, Malmgren L, Kristensson J, McGuigan FE, Akesson KE. In community-dwelling women frailty is associated with imminent risk of osteoporotic fractures. Osteoporos Int. 2021;32 (9):1735–44

**Table 2:** FRAX* by quartiles at age 75 and frailty score, fracture and falls

All variables at 75y	Low FRAX Q1 (<=19)		FRAX Q2 (20–26)		FRAX Q3 (27–33)		High FRAX Q4 (34+)		N^o^	*p*-value^#^ Overall	*p*-value Q1 vs Q4
	*n* = 303		*n* = 214		*n* = 279		*n* = 227				
	Mean	range	Mean	range	Mean	range	Mean	range			
**FRAX**_**MOF no BMD**_	16.9	(12–19)	22.6	(20 - 26)	30.0	(27–33)	41.8	(34–69)		–	–
Frailty index	0.15	(0.00–0.65)	0.16	(0.00–0.70)	0.16	(0.00–0.72)	0.19	(0.00–0.77)	1023	0.015	0.004
*Fractures*	N^o^	(%)	N^o^	(%)	N^o^	(%)	N^o^	(%)			
Prior any fx (20-75y)	30	(6.7)	48	(10.7)	187	(41.7)	183	(40.8)	448	< 0.001	< 0.001
Prior any fx (50-75y)	21	(5.6)	40	(10.7)	156	(41.7)	157	(42.0)	374	< 0.001	< 0.001
Prior MOF (50-75y)	19	(8.1)	23	(9.8)	101	(43.0)	92	(39.1)	235	< 0.001	< 0.001
*Falls in the last year*	N^o^	(%)	N^o^	(%)	N^o^	(%)	N^o^	(%)			
1 or more falls reported	66	(26.1)	45	(17.5)	75	(29.6)	67	(26.5)	253	0.118	0.057
3 or more falls reported	18	(27.7)	10	(15.4)	21	(32.3)	16	(24.6)	65	0.609	0.661
*Bone mineral density*	Median	IQR	Median	IQR	Median	IQR	Median	IQR			
Femoral neck T-score	−1.50	(1.65)	−1.80	(1.60)	−1.98	(1.48)	−2.34	(1.37)	946	< 0.001	< 0.001
Femoral neck BMD (g/cm^2^)	0.800	(0.200)	0.764	(0.192)	0.742	(0.178)	0.700	(0.164)	946	< 0.001	< 0.001

*FRAX_MOF no BMD_. Reported values are means, unless otherwise stated. ^#^ *p*-values calculated by ANOVA, Kruskal Wallace or Chi-square as appropriate

### Frailty in combination with FRAX for 10-year fracture probability

We wanted to investigate if frailty provided additional discriminative value to FRAX among older women. To compare the discriminative ability of the two measurements *individually,* separate ROC curves for frailty and FRAX were made. The ROC analyses indicated that for hip fracture, FRAX and frailty performed almost equally (HIP AUC 10y: 0.566 vs. 0.567, *p* = 0.015 vs *p* = 0.013). For perspective, the AUC for BMD was 0.639; *p* < 0.001. Next, FI was used *in conjunction* with FRAX; proving marginally better than either score alone (AUC 10y: 0.584, *p* = 0.002). These results were confirmed by Harrell’s C-statistic (HIP AUC 10y: FRAX: 0.589 vs FI: 0.604; combined 0.623).

Comparable results were observed for MOF, indicating that while FRAX alone was marginally better than frailty index, the combination added to the discriminative ability (MOF AUC 10y (FRAX and FI combined) 0.563; *p* = 0.001). Again for perspective, AUC for BMD was 0.618; *p* < 0.001 (MOF). Although frailty had a weak but significant correlation with FRAX_MOF, no BMD_ (r = 0.085; *p* = 0.007), no correlation between frailty and BMD was observed r = − 0.048; *p* = 0.142.

### The discriminative value of frailty in primary fracture prevention, i.e. within the non-treatment group

**Fig. 2:**
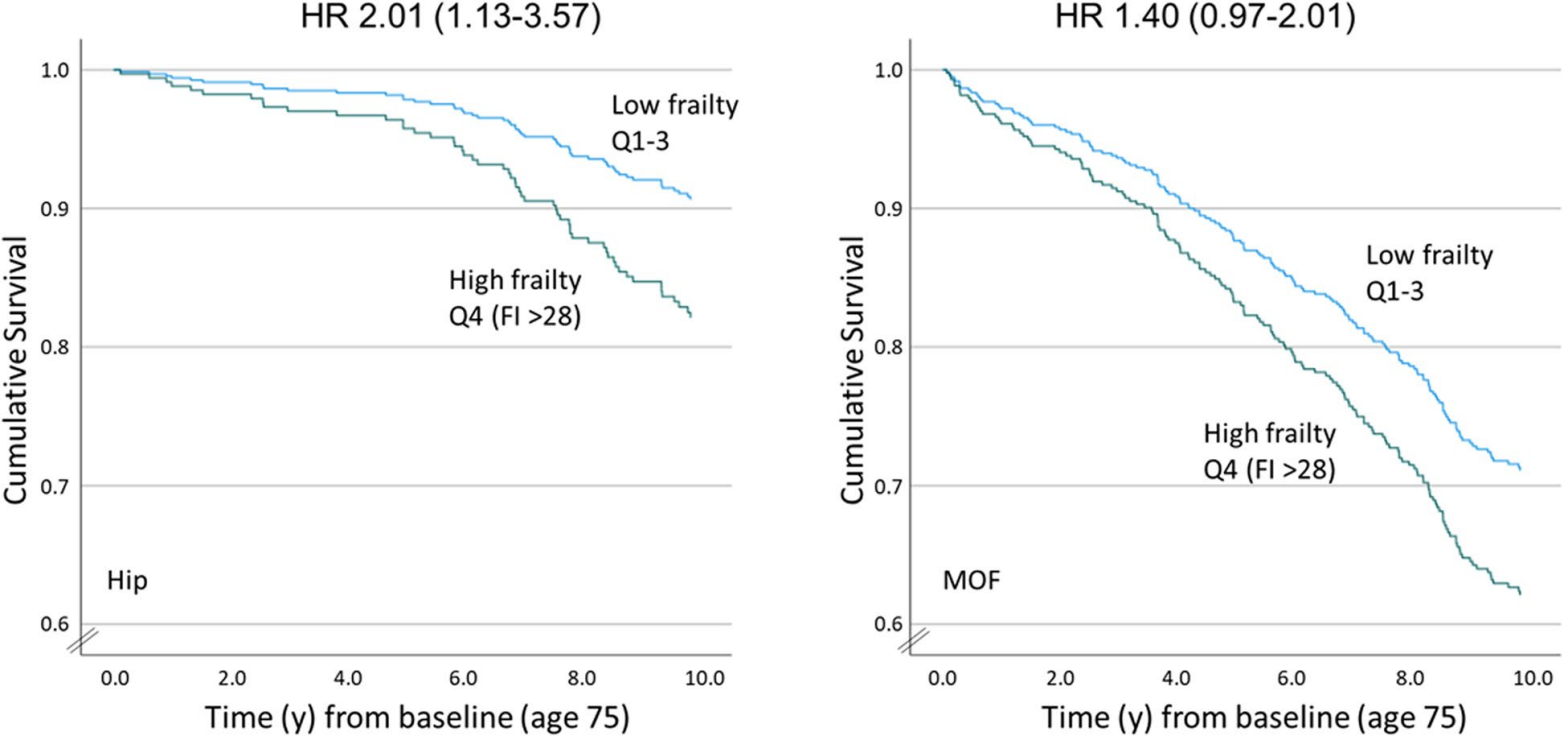
Fracture risk among women in the non-treatment group*, stratified by low and high frailty.* The non-treatment group was identified using the Swedish Osteoporosis Foundation and include those with no previous MOFs and either a FRAX_MOF_ < 15% or did not meet requirements for treatment after BMD measurement (i.e. T-score < − 2.5 and FRAX_MOF BMD_ > 20%). Low frailty includes Q1-Q3 (FI 0.0–0.27) and high frailty Q4 (FI > 0.28)

We sought to obtain a clinical perspective on the utility of assessing frailty for primary fracture management. Using guidelines from the Swedish Osteoporosis Society, a non-treatment group of 564 (54%) was defined. These women had not sustained a previous osteoporotic fracture and would not be recommended for pharmacological treatment based on FRAX_MOF_ or FRAX_MOF BMD_ in combination with T-score. To investigate the discriminative ability of FRAX and frailty in this ‘low risk’ non-treatment group, ROC curves were made. For hip fracture, while frailty alone could discriminate fracture (HIP AUC 10y: 0.590, *p* = 0.031), FRAX could not (HIP AUC 10y: 0.557, *p* = 0.169). The combination of frailty and FRAX did not improve discriminative ability compared to frailty alone (HIP AUC 10y: 0.585, *p* = 0.042). Neither frailty nor FRAX alone, or the combination of the two measurements could discriminate osteoporotic fractures (data not shown). The women in the non-treatment group were further categorised according to their frailty status where a FI ≥ 0.28 (corresponding to Q4) were considered frail and Q1-Q3 non-frail. Among the apparently ‘low fracture risk’ women in the non-treatment group, being frail was associated with a higher risk of hip fracture over 10 years (HR 2.01 (1.13–3.57)) compared to those who were considered not frail (Table 3, Fig. 2). However, this association did not reach statistical significance for osteoporotic fracture (HR 1.40 (0.97–2.01), frail vs non frail). In addition, at baseline the proportion of women reporting falls within the previous year in the non-treatment group was higher among those with high frailty (38.8% vs 22.1%; *p* = 0.002, data not shown).

**Table 3:** 10-year fracture risk for women in the non-treatment group* based on their frailty status (low vg high) at age 75

Frailty range	Non-treatment and LOW frailty*		Non-treatment and HIGH frailty*		Fracture Risk	
	(0.00–0.27) *n = 356*		(> 0.28) *n = 94*		HR (95% CI)	
*Fractures*						
N^o^ with Hip fx (10y)	37/443	(8%)	17/121	(14%)	2.01 (1.13–3.57)	*p* < 0.018
N^o^ with MOF (10y)	117/443	(26%)	39/121	(32%)	1.40 (0.97–2.01)	*p* < 0.072

*The non-treatment group was identified using the Swedish Osteoporosis Foundation and include those with no previous MOFs and either a FRAX_MOF_ < 15% or did not meet requirements for treatment after BMD measurement (i.e. T-score < − 2.5 and FRAX_MOF BMD_ > 20%). Low frailty includes Q1-Q3 and high frailty is Q4. Hazard ratios are unadjusted, comparing Q4 to Q1-Q3

The utility of measuring frailty was also demonstrated when using T-score to define fracture risk instead of FRAX. Among *n* = 678 non-osteoporotic women, frailty added to the 10-year risk of fracture (HR_HIP_ 2.22 (1.35–3.71) p = 0.002; HR_MOF_ 1.57 (1.15–2.14) *p* = 0.005, data not shown).

## Discussion

To capture more women at risk of fracture and to reduce the fracture burden, this study in 1023 community dwelling women investigated if frailty in conjunction with FRAX could improve fracture prediction over 10 years. Not only did the two measurements combined improve fracture prediction slightly, applying a frailty score to women at *apparent* low risk successfully identified a group with *actual* higher hip fracture risk in the non-treatment group. This is especially important since these women would not be prioritized for pharmacological treatment according to present guidelines. To prevent a primary facture in these individuals would not just decrease the fracture burden, but may very well prevent any further spiralling towards higher frailty. Taken together, our results suggest that there may be a clinical utility for measuring frailty in preventive fracture management.

The need to improve fracture management is urgent, not least due to the actual and expected increase of the economic burden [23]. Since a first fracture increases the risk by 86% of a further fracture [24], primary fracture prevention should be prioritized within this area. This study indicates that frailty could be of value in primary fracture prevention, identifying ‘at risk’ women without a previous osteoporotic fracture in the non-treatment group. Correspondingly, while frailty could discriminate hip fracture in the non-treatment group, FRAX could not. In addition, using FRAX in conjunction with the frailty index in this group did not improve discriminative ability compared to frailty alone. With a growing recognition of frailty as an important risk factor for adverse outcomes it is not unlikely that it also will become part of the standard assessment within the primary care setting [25], a part of the healthcare system often responsible for osteoporosis care. However, how to implement a measurement of frailty into fracture prevention is not clear, in part because there is as yet no general consensus of how to measure frailty.

A substantial part of the association between frailty and fracture risk is probably explained by the higher fall propensity among the frail women [6]. At present, there is ongoing work to optimize the FRAX algorithm, with the possibility of a future version including a history of falls, since the association between falls and fractures is widely recognized [26]. The initial reasons for *not including* falls into FRAX were that data on falls from the cohorts used to construct FRAX was not uniform, there was limited data on whether intervention against falls reduced fracture risk, and that more information was needed before falls could be incorporated into FRAX [27]. Moreover, it was considered that the risk of falling is implicitly captured without being measured directly [28]. However, in our specific setting this last assumption in not evident; when comparing quartiles of FRAX we fail to see any significant difference between falls within the previous year. We have previously tested which factors predict falls and found that a clinical history and a subjective estimate of a person’s health were more important than objective functional tests for fall prediction [29]. While most other recent studies in this area compare single risk factors for falls, frailty captures a wider spectrum of deficits.

Today, frailty is considered a dynamic and hence potentially reversible syndrome, but the current evidence supporting possible intervention to reverse or minimize the rate of decline into frailty are varied, as are the strategies employed [25, 30]. Based on this study, frailty could be an important tool to identify women at high risk of primary hip fracture. Should interventions for frailty prove successful, these may not only slow the trajectory into worsening frailty, but also prevent future first, and thus, subsequent fractures. While this is certainly an interesting field for future studies, it should also be noted that neither frailty, nor FRAX are perfect indicators of fracture suggesting there are still many, perhaps arbitrary, factors that affect an individual’s risk of fracture.

### Limitations and strengths

Limitations are acknowledged in this longitudinal study of fracture risk. Firstly, the number of women who have a low FRAX score and are also highly frail is relatively low. This is unavoidable in this age group, since at age 75 the majority of women are at high risk of fracture, and while data is promising, it requires testing in a larger sample size. A second limitation relates to generalisation, and applicability of the results to women at younger ages or to other ethnicities must be determined. Hence caution should be exercised in terms of generalising the findings. Thirdly, direct comparison with other studies may be difficult since a variety of frailty indices are used. Although a drawback, this reflects the lack of consensus in the present clinical situation. As a fourth limitation, participants in the OPRA cohort may be healthier than those who declined [31], which is a common phenomenon in older populations [32]. However, participation rate was high (65%), thus increasing the likelihood that the OPRA cohort is a representable sample of 75 year old women. Finally, we used ROC curves and AUC to evaluate the utility of frailty and FRAX. A recognized challenge with this methodology is that, for an existing model with fairly good discrimination, an additional risk factor is likely to give a very small change in the AUC, making *quantification* of its usability difficult. Nevertheless, it is clear that the addition of a frailty index does improve prediction of individuals at risk. This improvement may in part be explained by the strong association between frailty and falls [6].

Strengths of this study include that the participants are community dwelling, older women, who are at an age when osteoporotic fracture risk is high. Importantly, the longitudinal data for 10 years facilitates comparison with FRAX 10-year risk estimates, which are currently the recommended method of predicting fracture risk, but which do not incorporate frailty or falls as a clinical risk factor in the algorithm. Since all women were the same age at inclusion, there is reduced confounding from chronological age with respect to fracture risk and the accumulated health deficits captured by the frailty index. The relevance of this study lies in in demonstrating quantitatively that identifying vulnerable older women and targeting them for preventive fracture management, may be one important way of preventing fragility fractures in older women.

## Conclusions

In conclusion, while the addition of frailty to an existing prediction tool appears to marginally improve prediction of fractures, applying a frailty measurement to a group of ‘low risk’ women, identifies a set of individuals with higher *actual* hip fracture risk that would not be prioritized for pharmacological treatment according to present guidelines. Further cost-benefit analysis studies will be needed to formally test the potential benefit.

## Data Availability

The datasets used and/or analysed during the current study are available from the corresponding author on reasonable request.

## Abbreviations

AUC: Area Under the Curve
BMD: Bone Mineral Density
DXA: Dual-energy X-ray Absorptiometry
FI: Frailty Index
FRAX: Fracture Risk Assessment Tool
HR: Hazard Ratio
IQR: Interquartile Range
MOF: Major Osteoporotic Fracture
OPRA: Osteoporosis Risk Assessment Cohort
ROC: Receiver Operating Characteristic
SD: Standard Deviation
WHO: World Health Organization
